# Cardiovascular Disease Assessment Prior to Kidney Transplantation

**DOI:** 10.14797/mdcvj.1117

**Published:** 2022-09-06

**Authors:** Elise C. Ewing, Angelina R. Edwards

**Affiliations:** 1 Division of Renal Diseases and Hypertension, Houston Methodist Hospital, Houston, Texas, US; 2 Texas A&M College of Medicine, Houston, Texas, US

**Keywords:** cardiovascular screening, kidney transplantation, chronic kidney disease, end-stage kidney disease

## Abstract

Cardiovascular disease is highly prevalent and the leading cause of mortality in patients with chronic kidney disease, end-stage kidney disease, and kidney transplantation. However, kidney transplantation offers improved survival and quality of life, with an overall reduction in cardiovascular disease events; therefore, it remains the optimal treatment choice for those with advanced kidney disease. Pretransplantation cardiovascular assessment is performed prior to wait-listing and at routine intervals with the principal goal of screening for asymptomatic cardiac disease, intervening when necessary to improve long-term patient and allograft survival. Current clinical practice guidelines are based on expert opinion, with a lack of high-quality evidence to guide standardized screening practices. Recent studies support de-escalation in screening with avoidance of preemptive revascularization in asymptomatic patients, but they fail to provide clear guidance on how best to assess the cardiovascular fitness of this high-risk group. Herein we summarize current practice guidelines, discuss key study findings, highlight the role of optimal medical therapy, and evaluate future directions for cardiovascular disease assessment in this population.

## Introduction

Cardiovascular disease (CVD) is the leading cause of death for patients with advanced chronic kidney disease (CKD) and end-stage kidney disease (ESKD). When compared to maintenance dialysis, kidney transplantation offers improved survival and quality of life. However, the burden of CVD persists and is the leading cause of death with a functioning allograft.

Table 1 outlines traditional and nontraditional CVD factors in this population.^1^ All major types of CVD are represented in kidney transplant recipients, including coronary artery disease, heart failure, valvular heart disease, cerebrovascular disease, arrhythmias, and pulmonary hypertension. Decreased renal metabolic clearance leads to a uremic milieu with enhanced oxidative stress and proinflammatory cytokines. Hemodynamic changes lead to increased arterial wall stress and accelerated vascular calcifications. Hypervolemia leads to increased myocardial wall stress and can precipitate left ventricular hypertrophy, dysfunctional myocardial conduction, and pulmonary hypertension. Additionally, disorders of bone mineral metabolism and anemia of chronic kidney disease have been associated with a higher risk of CVD.

**Table 1:** Cardiovascular disease risk factors.^1^ CKD: chronic kidney disease; ESKD: end-stage kidney disease; LV: left ventricular


TRADITIONAL FACTORS	CKD/ESKD FACTORS	NONTRADITIONAL POSTTRANSPLANT FACTORS

Age	Anemia	Allograft dysfunction

Diabetes mellitus	Bone mineral metabolism	Chronic inflammation/oxidative stress

Dyslipidemia	Hypervolemia: LV hypertrophy and pulmonary hypertension	Hyperhomocysteinemia

Hypertension	Proteinuria	Metabolic consequence of immunosuppression

Physical inactivity	Uremic toxins	Posttansplant diabetes mellitus

Tobacco use	Vascular calcification	Obesity


Given the desire to maximize utility of scarce donor organs, the goals of pretransplantation cardiovascular risk stratification are (1) screening for asymptomatic coronary artery disease (CAD) or silent ischemic heart disease, (2) screening to determine optimal candidates for transplantation, and (3) screening to enhance long-term patient and allograft survival. Testing should appropriately identify those recipients who can withstand the perioperative surgical and anesthetic risk while excluding those with significant CAD burden that may lead to premature death.

The method of screening to provide risk prediction involves a combination of history and physical examination, blood tests, electrocardiograms, functional status evaluation, and a combination of noninvasive and invasive strategies. Modality for functional assessment of coronary heart disease varies by center expertise and local availability, while coronary angiography is limited by a candidate’s residual renal function. Recent studies of high-sensitivity troponin show that it may be a useful biomarker in patients with CKD and reflect not only myocardial injury but also other cardiovascular events, including stroke and peripheral arterial disease.^2,3^ Similarly, physical function status can provide key information on candidate fitness or frailty and is predictive of posttransplant outcomes.^4,5^ Broad use of these additional tests is limited by availability, and implementation varies from center to center.

Current screening guidelines are outlined in Table 2 and are based predominantly on expert opinion with minimal high-quality evidence.^6,7,8^ Although these guidelines exist, the approach to CVD assessment in the kidney transplant candidate varies greatly by provider and center. Cheng et al. surveyed transplant providers in the United States (US) in 2020 and found a heavy reliance on noninvasive testing with myocardial perfusion scintigraphy or dobutamine stress echocardiography and a predilection towards aggressive evaluation and revascularization of asymptomatic patients with abnormal stress test results.^9^ Similarly, Nimmo et al. surveyed transplant nephrologists in the United Kingdom in 2021 and discovered a wide variation in screening practices among the 23 kidney transplant centers, with all expressing concern about the lack of evidence upon which their practice is based.^10^ Randomized controlled trial evidence is not available to guide screening for asymptomatic CAD before transplantation. Moreover, a growing body of evidence consistently shows a lack of efficacy in demonstrating survival benefit in preemptive screening and intervention in asymptomatic patients and may deprive patients the opportunity to receive a transplant. The following highlights our current understanding of CAD in patients with advanced kidney disease and discusses the available evidence in kidney transplant candidates.

**Table 2:** Screening guidelines for cardiovascular disease in kidney transplant candidates based on recommendations from the 2012 American College of Cardiology/American Heart Association (ACC/AHA) Statement on Cardiac Disease Evaluation and Management among Kidney and Liver Transplantation Candidates, American Society of Transplantation-Kidney Pancreas Community of Practice (AST-KPCOP) Cardiovascular Disease Work Group, and Kidney Disease Improving Global Outcomes (KDIGO) 2020 Guidelines for the Evaluation of the Kidney Transplant Candidate.^6,7,8^ EKG: electrocardiogram; CVD: cardiovascular disease; CAD: coronary artery disease; LVEF: left ventricular ejection fraction; CT: computed tomography; CABG: coronary artery bypass grafting; PCI: percutaneous coronary intervention; ESRD: end-stage renal disease; RHC: right heart catheterization; OSA: obstructive sleep apnea; PAH: pulmonary artery hypertension; KT: kidney transplant; TAVR: transcatheter aortic valve replacement; NYHA: New York Heart Association Relevant risk factors among transplantation candidates include diabetes mellitus, prior cardiovascular disease, more than 1 year on dialysis, left ventricular hypertrophy, age greater than 60 years, smoking, hypertension, and dyslipidemia. The specific number of risk factors that should be used to prompt testing remains to be determined, but the committee considers three or more as reasonable.Significant pulmonary hypertension is defined as right ventricular systolic pressure more than 45 mm Hg on echocardiogram, or ancillary evidence of right ventricular pressure overload.Significant pulmonary arterial hypertension is defined by mean pulmonary artery pressure ≥ 25 mm Hg, pulmonary capillary wedge ≤ 15 mm Hg, and pulmonary vascular resistance of > 3 Wood units in the absence of an identified secondary cause (eg, obstructive sleep apnea, left heart disease).Risk factors for pulmonary hypertension included portal hypertension, connective tissue disease, congenital heart disease, and chronic obstructive pulmonary disease.


	ACC/AHA^6^	AST-KPCOP^7^	KDIGO^8^

Coronary Artery Disease	Preoperative 12-lead EKG in patients with known CVD or any cardiovascular symptoms (Class I, Level of Evidence C)		Evaluate all candidates for the presence and severity of cardiac disease with history, physical examination, and EKG (not graded).

	Preoperative 12-lead EKG in patients without known CVD or without any cardiovascular symptoms (Class IIa, Level of Evidence C)		

	Annual 12-lead EKG after listing (Class IIb, Level of Evidence C)		

	Noninvasive stress testing in candidates with no active cardiac conditions on the basis of multiple CAD risk factors^a^ regardless of functional status (Class IIb, Level of Evidence C)	Noninvasive testing is the preferred initial screening modality for CAD, including dobutamine stress echocardiography and myocardial perfusion imaging, although the predictive value of a positive noninvasive test for immediate posttransplant cardiovascular outcomes is unclear. Coronary angiography is a better predictor of posttransplant CVD-associated mortality, but the use of angiography is limited due to concerns about adverse events, especially renal injury in those not yet on dialysis.	Suggested that asymptomatic candidates at high risk for CAD (eg, diabetes, previous CAD), or with poor functional capacity undergo noninvasive CAD screening (2C).
	
	LVEF < 50%, evidence of ventricular chamber enlargement, exercise-induced hypotension, angina, or known ischemia should prompt referral to a cardiologist for management of ischemic heart disease (Class I, Level of Evidence B)		If any signs or symptoms of active cardiac disease, should undergo assessment by a cardiologist for further management prior to transplant (not graded).
	
	Uncertain role of noncontrast CT calcium scoring and/or cardiac CT angiography in pre-transplant risk stratification (Class IIb, Level of Evidence B)		Perform cardiac imaging in patients with systemic amyloidosis. If significant cardiac amyloid confirmed, recommend excluding such patients (not graded).

	Uncertain role of periodic screening for myocardial ischemia in asymptomatic listed candidates (Class IIb, Level of Evidence C)	Once evidence of ischemic heart disease (typically by noninvasive cardiac stress testing) is found in the potential kidney transplant candidate, careful serial cardiovascular assessment must continue during wait-list time.	Suggested that candidates with myocardial infarction be assessed by a cardiologist to determine whether further testing is warranted and when to safely proceed with kidney transplant (2B). Suggest that transplant be delayed an appropriate amount of time after placement of a coronary stent based on cardiologist recommendation (2B).

	CABG is preferred to PCI in kidney transplant candidates with multivessel CAD and diabetes mellitus (Class IIa, Level of Evidence B).	Large prospective randomized studies will be needed to determine the efficacy of preoperative coronary revascularization on posttransplant cardiovascular outcomes.	Suggested that patients with asymptomatic, advanced triple-vessel CAD be excluded from kidney transplant unless they have an acceptable estimated survival (2D).

	Prophylactic revascularization in patients with stable CAD that will not improve symptoms or survival is not recommended prior to transplant surgery (Class III, Level of Evidence B).	It remains to be determined if preoperative risk stratification and ultimately revascularization, when indicated, will improve cardiovascular outcomes following kidney transplant.	Recommend that asymptomatic candidates with known CAD not be revascularized exclusively to reduce perioperative cardiac events (1B).

Heart failure	Reasonable to perform preoperative echocardiographic assessment of LV function in potential kidney transplant candidates (Class IIa, Level of Evidence B).	Larger studies are needed to define the incremental predictive value of clinical and echocardiographic parameters (including global longitudinal strain) for adverse CVD events in kidney transplants.	Suggested that patients with uncorrectable, symptomatic NYHA Class III/IV heart disease be excluded from kidney transplant unless there are mitigating factors that give the patient an acceptable estimated survival (2D). Assess with cardiologist and consider combined/simultaneous heart and kidney transplant.

Valvular disease	Consider yearly echocardiogram in ESRD patients with moderate aortic stenosis (Class IIb, Level of Evidence C).	Outcomes among patients with KT undergoing TAVR versus open surgical replacement have only been examined in retrospective analyses, with variable outcomes reported. Larger studies will be needed to identify more reliable estimates of outcomes following TAVR in KT recipients.	Patients with severe valvular heart disease should be evaluated and managed by a cardiologist according to local cardiac guidelines (Not graded)

Pulmonary hypertension^b^	Reasonable to evaluate for secondary causes (OSA, left heart disease)(Class IIa, Level of Evidence C).	While RHC is the gold standard for the diagnosis of PH, transthoracic echocardiography is the most commonly used technique to assess pulmonary pressures in practice, given the expensive and invasive nature of RHC.	Suggested that asymptomatic candidates who have been on dialysis for at least 2 years or have risk factors for pulmonary hypertension^d^ undergo echocardiography (2D).
	
	Consider RHC to confirm echocardiographic evidence of elevated PA pressures (Class IIb, Level of Evidence C).		Recommend not excluding candidates with uncorrectable pulmonary artery systolic pressure > 60 mm Hg by RHC, but consider the risks of sudden deterioration or progression after transplant, and patient should have an acceptable estimated survival (1C).

	If RHC confirms significant PAH,^c^ consider referral to a pulmonary vascular disease specialist (Class IIa, Level of Evidence C).	There is demonstrated importance in closely managing pulmonary hypertension preoperatively.	Patients with estimated pulmonary systolic pressure > 45 mm Hg by echo should be assessed by a cardiologist (not graded).


## Stable Ischemic Heart Disease in Patients with Advanced Kidney Disease

Historically, studies of ischemic heart disease largely excluded patients with advanced CKD or ESKD, and management of this population was based on extrapolation and low-quality evidence from observational studies; however, recent trials have allowed a more objective assessment (Table 3).^11,12,13,14,15,16,17^

**Table 3:** Major trials of patients with ischemic heart disease treated with percutaneous coronary intervention (PCI), revascularization with coronary artery bypass graft (CABG), and/or conservative optimal medical therapy (OMT).^11,12,13,14,15,16,17^ COURAGE: Clinical Outcomes Utilizing Revascularization and Aggressive Drug Evaluation; DIAD: Detection of Ischemia in Asymptomatic Diabetics; BARI-2D: Bypass Angioplasty Revascularization Investigation 2 Diabetes; FAME 2: Fractional Flow Reserve versus Angiography for Multivessel Evaluation 2 Trial; ISCHEMIA: International Study of Comparative Health Effectiveness with Medical and Invasive Approaches; ISCHEMIA-CKD: International Study of Comparative Health Effectiveness with Medical and Invasive Approaches-Chronic Kidney Disease; CAD: coronary artery disease; DM: diabetes mellitus; CKD: chronic kidney disease; eGFR: estimated glomerular filtration rate; PCI: percutaneous coronary intervention; OMT: optimal medical therapy; CABG: coronary artery bypass graft; FFR: fractional flow reserve; MI: myocardial infarction: CV: cardiovascular


YEAR/TRIAL	PATIENT CHARACTERISTICS	RENAL FUNCTION			INTERVENTION	FOLLOW-UP PERIOD	PRIMARY OUTCOME	OVERALL EFFECT

		eGFR <60	eGFR <30	DIALYSIS				

2007/COURAGE^11,12^	2,287 patients, stable CAD	320 (14%)	16 (0.7%)	None, excluded from the study	PCI + OMT vs OMT alone	4.6 years	All-cause death; nonfatal MI	No difference in primary outcome

2009/DIAD^17^	1,123 patients, Type 2 DM, no symptoms of CAD	None	None	None, excluded from the study	561 patients with adenosine sestamibi MPI vs 562 patients no screening	4.8 years	All-cause death; nonfatal MI	No difference in primary outcome in those screened

2009/BARI-2D^13^	2,368 patients, type 2 diabetes & stable CAD	494 (21%)	None, excluded from study	None, excluded from study	PCI+ OMT or CABG + OMT vs OMT alone	4.4 years	All-cause death, MI, or stroke	No difference in primary outcome

2012/FAME 2^14^	888 patients	22 (2%)	None, excluded from study	None, excluded from study	FFR (< 0.8)-guided PCI + OMT vs OMT alone	5.04 years	All-cause death, MI or urgent revascularization	No difference for FFR > 0.8 on OMT

2020/ISCHEMIA^15^	5,179 patients, stable CAD and moderate or severe ischemia	568 (11%)	None, excluded from study	None, excluded from study	Initial invasive PCI or CABG + OMT vs conservative OMT + revascularization as needed	3.2 years	CV death, nonfatal MI, hospitalization for unstable angina, heart failure, cardiac arrest with resuscitation	No difference in primary outcome

2020/ISCHEMIA-CKD^16^	777 patients, advanced CKD and moderate to severe ischemia on stress	None	362 (47%)	415 (53%)	Initial invasive PCI or CABG + OMT vs conservative OMT + revascularization as needed	2.2 years	All-cause death or nonfatal MI, hospitalization for unstable angina, heart failure or cardiac arrest with resuscitation	No difference in primary outcome; increased incidence of new onset dialysis and stroke


Optimal management of CAD in the CKD population was assessed by Bangalore et al. in the International Study of Comparative Health Effectiveness with Medical and Invasive Approaches - Chronic Kidney Disease (ISCHEMIA-CKD) trial. A total of 777 patients with advanced kidney disease (eGFR < 30 mL/min/1.73 m^2^ or dialysis dependence) and moderate or severe ischemia on stress testing were randomized to either treatment with an invasive strategy of coronary angiography and revascularization (if indicated) with medical therapy, or a conservative strategy with medical therapy and subsequent angiography in those whom medical therapy failed. Contrary to the original expectation, the invasive strategy did not confer cardioprotective benefit, with no difference observed between the primary outcomes of 3-year event rate of death or nonfatal myocardial infarction (MI) (HR 1.01; 95% CI, 0.79-1.29; *P* = .95). Furthermore, the invasive strategy was associated with a 3.76-fold higher hazard ratio of stroke and 48% higher hazard ratio of death or initiation of dialysis, likely related to atheroembolic complications of coronary angiography and revascularization (HR 1.48; 95% CI,1.04-2.11; *P* = .03).^16,18^

## Management Approach to Stable Ischemic Heart Disease in the Kidney Transplant Candidate

Since CVD is the most common cause of death with a functioning graft, transplant candidates are routinely screened for asymptomatic coronary artery disease. However, the historic support for preemptive revascularization of transplant candidates has been based on observational studies and one small randomized controlled trial. Manske et al. published their findings in 1992 of 26 wait-listed insulin-dependent diabetic patients who were kidney transplant candidates with asymptomatic CAD. Half of the patients were randomized to medical management (calcium channel blocker vs aspirin) and the other half to revascularization (angioplasty or coronary artery bypass surgery). Of the 13 who were medically managed, 10 had a cardiovascular event within a median of 8.4 months compared to only 2 of the 13 revascularized patients. Unfortunately, despite its small size and conduct in a different treatment era, this became the basis for routine angiography for diabetic renal transplant candidates with revascularization for symptomless stenosis.^18^

In 2004, McFalls and colleagues in the Coronary-Artery Revascularization before Elective Major Vascular Surgery (CARP) trial assessed long-term outcomes of 510 patients with clinically significant CAD (> 70% stenosis) scheduled for elective vascular surgery (either repair of expanding abdominal aortic aneurysm or peripheral arterial disease), randomized to pre-op revascularization, or none. Median time between randomization and elective surgery was 36 days longer in the revascularization group. There was no difference between 30-day post-elective procedure MI or mortality at 2.7 years between the two groups. Medical therapy was optimized in both groups, with utilization rates of beta-blockers exceeding 80%, statin use of 50%, and aspirin use of 70%.^19^ This questioned the practice of routine revascularization, particularly in patients undergoing high-risk vascular surgeries, for which kidney transplant could be considered equivalent.

Most recently, an important post hoc analysis of the ISCHEMIA-CKD trial argues against routine revascularization for stable kidney transplant candidates. Herzog and colleagues evaluated 194 of 777 transplant candidates with chronic coronary syndrome and at least moderate ischemia on myocardial perfusion scan. Compared to nonlisted patients, listed patients were younger (60 years vs 65 years) and more likely to be on dialysis (85% vs 44%), had less anginal symptoms, and were more likely to receive angiography regardless of treatment assignment. Among patients assigned to an invasive approach, the adjusted hazard ratio for the primary outcome was 0.91 and 1.03 (*P* = .68) for those listed and not listed, respectively. The 3-year cumulative incidence of major adverse cardiovascular events (MACE) did not differ based on intervention, with rates of 29% in the invasive strategy and 30% in the conservative strategy. An invasive strategy with preemptive revascularization compared with conservative optimal medical therapy (OMT) did not improve all-cause mortality or nonfatal MI in these patients. Surprisingly, nonprotocol-specified angiography was performed in one-third of listed patients in the conservative strategy, with 20% of them receiving revascularization (ie, crossover strategy). This may have impacted the potential difference in outcome but more importantly points to the persistent lingering concern of clinicians regarding nonintervention on waitlisted patients with evidence of inducible moderate or severe myocardial ischemia.^20^

A recent meta-analysis by Siddiqui et al. evaluated eight studies, predominantly retrospective and prospective cohort studies with one randomized control study, with 945 wait-listed kidney transplant candidates with established CAD. No difference was found in all-cause mortality, cardiovascular mortality, and MACE, including MI, acute coronary syndrome, heart failure, or ventricular arrythmias in those managed with coronary revascularization compared to OMT prior to transplantation.^21^ In keeping with established practice guidelines (as noted in Table 2), asymptomatic kidney transplant candidates with known CAD should not undergo routine coronary revascularization exclusively to reduce perioperative events. Siddiqui and colleagues conclude that OMT should be pursued in asymptomatic patients and revascularization should be reserved for those with high-risk anatomic subsets where intervention would allow improved survival. While these trials cast doubt on the utility of routine angiography and percutaneous coronary intervention solely for improving outcomes in wait-listed patients with CKD, they do not specifically address the optimal screening strategy in kidney transplant candidates.

## Impact of Pretransplant Screening and Posttransplant Cardiovascular Outcomes

Routine screening of the kidney transplant candidate occurs at the time of wait-listing and at regular intervals until transplantation. It would follow that this pretransplant screening should improve posttransplant cardiac outcomes in those candidates with asymptomatic CAD. However, this is not the case. Nimmo et al. conducted a national prospective study of 2,572 kidney transplant recipients assessing if pretransplant screening with stress test or coronary angiogram led to any difference in MACE up to 5 years posttransplant. Reassuringly, incidence of MACE was low (0.9% at 90 days, 2.1% at 1 year, 9.4% at 5 years), and no statistically significant association was observed between screening for asymptomatic CAD by angiography or stress test and MACE. These findings suggest that most of these transplant candidates were deemed acceptable cardiac risk, but it raises a question of whether others who may benefit from the superior treatment option of a transplant were unnecessarily excluded. It also highlights the possibility of a different etiology for cardiovascular disease in those with advanced CKD and posttransplant MACE.

Over 50% of cardiovascular deaths in transplant recipients are related to dysrhythmias rather than atherosclerotic events and are likely due to the high prevalence of systolic and diastolic dysfunction, left ventricular hypertrophy, myocardial stunning and fibrosis, electrical instability, and extraosseous vascular calcifications observed in these patients. More should be done to address the electrolyte and volume derangements that directly correlate with cardiac events, in particular sudden cardiac death.^22^ Current screening practices that focus only on atherosclerotic burden fail to fully assess cardiac risk and may be harmful or wasteful, leading to delays in listing, unnecessary exposure to ionizing radiation, loss of residual kidney function, and ultimately exclude patients inappropriately labeled as “high risk” from proceeding with a life-saving transplant.

## Challenges of Optimal Medical Therapy in Advanced Kidney Disease

The medical management of stable ischemic heart disease patients with advanced kidney disease should follow that of the general population. Emphasis on physical activity, dietary restriction of saturated fat, and tobacco cessation are particularly challenging but feasible interventions. In patients with ESKD, anemia management and optimization of bone mineral metabolism abnormalities are also necessary. Existing standards of medical management are summarized in Table 4.^23,24,25,26,27,28^

**Table 4:** Guideline recommendations for optimal medical therapy to address major CVD risk factors in patients with advanced kidney disease.^23,24,25,26,27,28^ BP: blood pressure; Hb: hemoglobin; HD: hemodialysis; HTN: hypertension; CKD: chronic kidney disease; DASH: Dietary Approaches to Stop Hypertension; PD: peritoneal dialysis; T2DM: type 2 diabetes mellitus; eGFR: estimated glomerular filtration rate; MI: myocardial infarction; *SGLT2i*: sodium/glucose cotransporter-2 inhibitors; ESAs: erythropoiesis-stimulating agents; HD: hemodialysis; PD: peritoneal dialysis; PTH: parathyroid hormone


TRADITIONAL CVD RISK FACTORS	

Hypertension^23,25^	Ambulatory BP monitoring should be used to complement standardized office BP readings (2B) Target sodium intake < 2 g daily in patients with HTN and CKD (2C); use caution in recommending DASH diet to CKD patients given risk of hyperkalemia Advise at least 150 minutes per week of moderate-intensity physical activity (2C) Target systolic blood pressure (SBP) of < 120 mm Hg when tolerated (2B) Recommend starting renin-angiotensin-system inhibitors (angiotensin-converting enzyme inhibitor [ACEI] or angiotensin II receptor blocker [ARB]) for patients with HTN, CKD, and moderate-to-severe albuminuria, with or without diabetes (grade varies based on degree of albuminuria and presence or absence of diabetes) Recommend avoiding any combination of ACEI, ARB, and direct renin inhibitor in patients with CKD (1B)

Dyslipidemia^26^	In adults with newly identified CKD (including those treated with chronic dialysis or kidney transplantation), recommend evaluation with a lipid profile (1C); follow-up measurement of lipid levels is not needed for majority of patients In adults aged ≥ 50 years with CKD and eGFR ≥ 60 mL/min/1.73m^2^ (stage 1-2), recommend treatment with a statin (1B) In adults aged ≥ 50 years with eGFR < 60 mL/min/1.73m^2^ (stage 3a-5) but not treated with chronic dialysis or kidney transplantation, recommend treatment with a statin or statin/ezetimibe combination (1A) In adults aged 18-49 years with CKD but not treated with chronic dialysis or kidney transplantation, statin therapy is suggested in people with one or more of the following: known coronary disease (MI or coronary revascularization), diabetes mellitus, prior ischemic stroke, estimated 10-year incidence of coronary death or nonfatal MI > 10% (2A) In adults with dialysis-dependent CKD, it is suggested that statins or statin/ezetimibe combination not be initiated (2A) but can be continued if the patient was already receiving statin or statin/ezetimibe combination at the time of dialysis initiation (2C) In adults with hypertriglyceridemia and CKD (including those on chronic dialysis and kidney transplant), suggested to advise therapeutic lifestyle changes (2D)

Diabetes mellitus^24^	Recommended to use hemoglobin A1c (HbA1c) to monitor glycemic control in patients with diabetes and CKD (1C) Recommend an individualized HbA1c target ranging from < 6.5% to < 8.0% in patients with diabetes and CKD not treated with dialysis (1C) Recommend lifestyle modifications including a well-balanced diet and moderate-intensity physical activity (1D) Suggested to maintain a protein intake of 0.8 g protein/kg/day for those with diabetes and CKD not treated with dialysis (2C); suggested to limit sodium intake to < 2 g per day (2C) Recommend treating patients with T2DM and CKD with eGFR ≥ 30 mL/min/1.73m^2^ with metformin (1B) and a sodium-glucose cotransporter-2-inhibitor (SGLT2i) (1A) If glycemic target not achieved with metformin and SGLT2i, or if unable to use those medications, recommended to treat with a long-acting glucagon-like peptide-1 receptor agonist (GLP-1 RA) (1B) and additional drug therapy as needed Recommend treatment with an ACEi or ARB be initiated in patients with diabetes, HTN, and albuminuria and titrated to the highest approved tolerated dose (1B)

**CKD / ESKD FACTORS**	

Anemia^27^	Anemia in adults with CKD is defined as Hb < 13 g/dL in men and < 12 g/dL in women Guidelines provide specific recommendations on iron supplementation and ESAs in this population Goal should be to maintain Hb level between 10-12 g/dL as many studies have consistently shown better outcomes in HD, PD, and pre-dialysis patients, without an increase in adverse reactions

Hypervolemia	Dry weight reduction in hypertensive hemodialysis patients is associated with improved BP control Per the DRIP trial, a post-dialysis weight reduction of 0.9 kg after 4 weeks was associated with a systolic BP reduction of -6.9 mm Hg (95% CI, -12.4 to -1.3 mm Hg; *P* = .016), and diastolic BP reduction of -3.1 mm Hg (95% CI, -6.2 to -0.02 mm Hg; *P* = .048)^26^ Reducing dry weight was generally well tolerated, but did result in an increase in intradialytic signs and symptoms of hypotension Lung ultrasound-guided strategy may predict which patients can safely benefit from dry weight reduction to target reduced ambulatory blood pressure levels ^27^

Disorders of mineral metabolism^28^	Patients with CKD 3a-5D should undergo serial assessments of phosphate, calcium, and PTH levels In patients with CKD 3a-5D, it is suggested to lower elevated phosphate levels toward the normal range (2C) and avoid hypercalcemia (2C) In patients with CKD 3a-5D with hyperphosphatemia, it is suggested to limit dietary phosphate intake alone or in combination with other treatments (2D). If receiving phosphate-lowering treatment, it is suggested to restrict the dose of calcium-based phosphate binders (2B) In patients with CKD 3a-5 not on dialysis, optimal PTH level is not known; it is suggested that patients with persistently high or progressively rising intact PTH levels should be evaluated for modifiable factors of hyperphosphatemia, hypocalcemia, high phosphate intake, and vitamin D deficiency (2C) In patients with CKD 4-5 calcitriol and vitamin D analogs should be used for progressive hyperparathyroidism (2C) In dialysis patients requiring PTH-lowering therapy, it is suggested to use calcimimetics, calcitriol, or vitamin D analogs, or a combination (2B)


The vast majority of pre-kidney transplant candidates are hypertensive. Recommendations on hypertension management in nondialysis-dependent CKD patients are summarized in the Kidney Disease Improving Global Outcomes (KDIGO) guidelines and are largely informed by data from the Systolic Blood Pressure Intervention Trial (SPRINT).^23,29^ Despite its exclusion of advanced CKD (eGFR < 20 mL/min) and all ESKD patients, it remains the largest randomized controlled trial for this population. An intensive blood pressure goal of systolic < 120 mm Hg significantly reduced the rates of adverse cardiovascular events and all-cause mortality. When considering the optimal guideline-based antihypertensive regimen, renin-angiotensin-aldosterone system (RAAS) blockade via angiotensin-converting enzyme inhibitors (ACEIs) or angiotensin receptor blockers (ARBs) along with beta-blockers in heart failure remains a cornerstone for patients with advanced kidney disease. However, both the achievement of these blood pressure targets and the use of these medications remains suboptimal.

Observations from the ISCHEMIA-CKD trial showed that at the time of enrollment, less than 50% of patients had systolic blood pressure < 140 mm Hg, and over 54% were not on RAAS blockade.^16^ While diminished renal function and hyperkalemia may be observed with RAAS blockade, aggressive use of these agents needs to be pursed. A recent review by Qiao et al. found that discontinuation of ACEIs or ARBs in patients once eGFR < 30 mL/min was associated with a higher risk of MACE and mortality within 5 years of discontinuation compared with those who remained on them; however, they saw no statistically significant difference in the risk of ESKD. Therefore, continuation of RAAS inhibition therapy in patients with declining kidney function may be associated with cardiovascular benefit without excessive harm of ESKD.^30^ Management of blood pressure in ESKD patients must focus on maintenance of euvolemia in addition to antihypertensive agents.^31,32^

High-intensity statin use has proven to be a safe and effective way to reduce CVD risk, but they are underused in the CKD population. Mefford et al. assessed statin use among US adults with CKD between 1999 and 2014 and found that over 65% of adults with CKD met the indication for statins based on the 2013 American College of Cardiology/American Heart Association (ACC/AHA) cholesterol guidelines, yet only 35.7% were taking a statin between 2011 and 2014.^33^ Lipid management in patients with ESKD remains controversial due to conflicting evidence.

The impact of statin therapy for reduction of MACE in ESKD patients was assessed in the 4D Study (Randomized Controlled Trial on the Efficacy and Safety of Atorvastatin in Patients with Type 2 Diabetes on Hemodialysis), AURORA (Rosuvastatin and Cardiovascular Events in Patients Undergoing Hemodialysis), and SHARP (Study of Heart and Renal Protection) trials.^34,35,36^ In both the 4D Study and AURORA trial, statin use compared to placebo resulted in a significant reduction in low-density lipoprotein (LDL) cholesterol but no reduction in the primary outcomes of cardiovascular death, nonfatal MI, or stroke. The SHARP trial, however, showed a possible benefit in lowering the incidence of atherosclerotic cardiovascular events with the combination of simvastatin and ezetimibe. Several meta-analyses and post-hoc analyses have continued to demonstrate discordant results, but the overall perception is that statins may be associated with decreased all-cause and cardiovascular mortality in this population. In the posttransplant setting, KDIGO guidelines recommend that all kidney transplant recipients be treated with a cholesterol-lowering agent regardless of LDL level, based on evidence from the Assessment of Lescol in Renal Transplantation (ALERT) trial.^37,38^

Strict glycemic control is an important factor in medical management of CKD patients with diabetes. Recent guidelines strongly support the use of SGLT2-inhibitors after multiple studies have shown benefit in glycemic control along with reduction in CVD risk and CKD progression.^24^ These medications remain underutilized—as low as 32% in eligible patients with CKD and type 2 diabetics—largely due to cost and patient or physician preference.^39^ A collaborative approach among primary care providers, nephrologists, endocrinologists, and cardiologists should be pursued to enhance use.

## Optimal Frequency of Cardiovascular Screening in Stable Wait-Listed Transplant Candidates

While living kidney donation allows a predictable timeline to transplantation, wait times on the deceased donor kidney transplantation list are prolonged, with less than half transplanted within 5 years of listing and variable transplant rates based on donation service area and geographic location.^40^ Wait-list maintenance and transplant candidate readiness remain challenging tasks for transplant centers, accounting for the rising trend in kidney transplantation costs.^41^ Despite recommendations by ACC/AHA that periodic screening of asymptomatic patients is uncertain, the current standard of care remains screening at regular intervals, either annually for diabetic patients or every 2 to 3 years for all other wait-listed patients. It is unclear whether eliminating this annual testing in asymptomatic candidates is noninferior to continued CVD screening for the primary prevention of MACE. The Canadian-Australian Randomized Trial of Screening Kidney Transplant Candidates for Coronary Artery Disease (CARSK; NCT03674307) study will test the hypothesis that eliminating screening for asymptomatic candidates is noninferior to annual testing.^42^ Additionally, a modeled cost-utility analysis of Australian and New Zealand kidney transplant candidates on the wait list by Ying et al. demonstrated an incremental cost-effectiveness ratio of $11,122 per quality-adjusted life year gained, with no screening compared to regular screening and a survival advantage of 0.49 life-year with no further screening.^43^ These trials will provide critical information to guide transplant centers on the optimal assessment and management of transplant candidates.

## Conclusion

Patients with advanced chronic kidney disease carry a high burden of cardiovascular risk factors, which lead to increased mortality both before and after transplantation. The optimal modality for screening and management of ischemic heart disease is unclear, and testing should not be overlooked for other cardiac pathology, such as valvular disease, diastolic dysfunction, and dysrhythmias. Current screening guidelines are based on moderate-quality evidence. Given the lack of survival benefit with preemptive screening and intervention in asymptomatic patients and the elevated risk of harm, de-escalation of cardiac screening appears warranted. The focus of current practice guidelines suggests that asymptomatic kidney transplant candidates should receive optimal medical therapy, with revascularization reserved for high-risk subsets. Additional studies are needed to guide a standardized evidence-based approach to screening that balances precision and resource utilization.

## Key Points

- There is no convincing evidence that preemptive revascularization in pretransplant candidates with stable coronary artery disease improves posttransplantation outcomes.
- Optimal medical therapy and meeting dialysis metrics remain the foundation for management of asymptomatic cardiovascular disease in patients with advanced kidney failure.
- Additional tools, such as physical performance testing and biomarkers, require more careful study and may aide in the assessment of cardiac fitness in kidney transplant candidates.
